# A Case of Necrotizing Periodontitis in a Care-Requiring Elderly Person Treated and Managed by Interprofessional Collaboration

**DOI:** 10.3390/dj10050079

**Published:** 2022-05-07

**Authors:** Masahiko Okubo, Ryutaro Kuraji, Hideyuki Kamimura, Yukihiro Numabe, Ko Ito, Tsuyoshi Sato, Shoichiro Kokabu

**Affiliations:** 1 Department of Oral and Maxillofacial Surgery, Faculty of Medicine, Saitama Medical University, Iruma 350-0495, Japan; masahiko.okb@sdnh.jp (M.O.); itok1127@saitama-med.ac.jp (K.I.); tsato@saitama-med.ac.jp (T.S.); 2 Kamimura Dental Orthodontic Clinic, Koshigaya 343-0823, Japan; hideksummer@gmail.com; 3 Department of Periodontology, School of Life Dentistry at Tokyo, The Nippon Dental University, Tokyo 102-8159, Japan; r-kuraji@tky.ndu.ac.jp (R.K.); numabe-y@tky.ndu.ac.jp (Y.N.); 4 Division of Molecular Signaling and Biochemistry, Kyushu Dental University, Kitakyushu 803-8580, Japan

**Keywords:** necrotizing periodontitis, necrotizing periodontal diseases, nursing care, direct oral anticoagulant, interprofessional collaboration

## Abstract

Background: Necrotizing periodontitis (NP) is a reactive and destructive inflammatory process that occurs in response to bacterial infection. Predisposing factors such as compromised host immune responses contribute significantly to NP pathogenesis. NP occasionally progresses to a more advanced and life-threatening state. Case presentation: A 73-year-old man in need of nursing care visited our dental clinic with severe gingival pain and intraoral bleeding. He had a disability and was immunocompromised because his medical history included cerebral infarction and type 2 diabetes mellitus. He was diagnosed with NP based on his typical symptoms, such as prominent bleeding and suppurative discharge from the gingiva, in addition to crater-shaped ulcerations of the interdental papillae. To improve daily oral hygiene, periodontists, dentists, and dental hygienists educated care workers and other staff at the nursing home on appropriate oral cleansing, including brushing three times a day using the Bass technique. Basic periodontal therapy, including whole-mouth scaling and debridement of the root surfaces using hand and ultrasonic instruments, was also performed. After this basic treatment of NP, we extracted the hopeless teeth. Currently, dentists visit the patient fortnightly to manage his oral hygiene. To date, good oral health has been maintained.

## 1. Introduction

Society is rapidly aging not only in developed countries but also in developing countries. The risk of severe periodontitis increases with age because of difficulties in plaque control due to disability or immunocompromise. Many elderly patients do not receive adequate dental treatment because they lack awareness of the disease or have difficulty accessing healthcare. Sufficient dental management in aging patients is necessary.

Necrotizing periodontitis (NP), previously referred to as necrotizing ulcerative periodontitis (NUP), is characterized by pain, bleeding, and ulceration of the gingival interdental papilla [1]. In the 1999 classification, necrotizing ulcerative gingivitis (NUG) and NUP were included among necrotizing periodontal diseases (NPD) as necrotizing gingivitis (NG) and NP, respectively [2]. NPD is a reactive and destructive inflammatory process that develops in response to bacterial infections, including those due to spirochetes and fusiform bacteria, and represents the most severe form of biofilm-related oral diseases [3]. NPD occasionally progresses to a more advanced and life-threatening state, namely, noma [4,5]. Although NPD is an infectious condition, immunosuppression is the most critical predisposing factor for it. NPD is almost always found in patients with HIV/AIDS but is also found in other conditions, such as leukemia, neutropenia, diabetes, and long-term immunosuppressant therapy [6,7,8,9]. Other etiological factors are tobacco smoking, malnutrition, psychosocial stress, poor oral hygiene, and poor sleeping habits [10]. Older adults are predicted to be immunosuppressed; however, epidemiological information regarding NPD in the elderly population remains unknown.

Thus, the most significant predisposing factor, such as compromised host immune response, significantly contributes to NPD pathogenesis. Therefore, in the 2018 classification, NPD was largely classified into two categories based on host immune response impairment. The first is that which occurs in severely chronically immunocompromised patients with systemic conditions, such as HIV/AIDS, severe malnutrition, severe infections, or extreme living conditions. In contrast, the second type occurs in transiently or moderately compromised patients with psychosocial stress, mild malnutrition, or smoking status. In addition, the current classification states that the pathological feature of NPD is ulcers in the stratified squamous epithelium and acute inflammatory cell infiltration in the gingival connective tissue. These findings clearly distinguish NPD from other periodontal diseases [11]. 

Here, we report a case of NPD in a compromised elderly man in need of nursing care who had a good outcome with adequate treatment and maintenance using a team approach.

## 2. Case Presentation

A 73-year-old man in need of nursing care visited our dental clinic in 2020 with severe gingival pain and intraoral bleeding. His medical history included cerebral infarction, hypertension, atrial fibrillation, and type 2 diabetes mellitus. The patient was treated with apixaban (10 mg/day), nifedipine (30 mg/day), olmesartan medoxomil (20 mg/day), metformin hydrochloride (75 mg/day), sitagliptin phosphate hydrate (15 mg/day), lansoprazole (10 mg/day), flunitrazepam (1 mg immediately before sleep), zolpidem tartrate (5 mg immediately before sleep), and sennosides (12 mg immediately before sleep). The patient’s family medical history was unremarkable.

### 2.1. Day 1: First Examination

**Figure 1:**
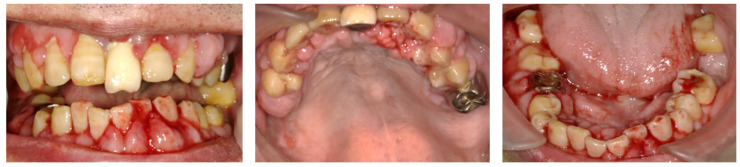
Intraoral photos taken at the patient’s first visit reveal generalized gingival redness, swelling, and bleeding.

At the first examination, the patient’s gingiva showed generalized redness and bleeding with spontaneous pain (Figure 1). Prominent bleeding and discharge of suppuration were observed in the gingiva. The interdental papillae exhibited crater-shaped ulcerations. Severe oral halitosis was present. Physical examination findings were as follows: body temperature, 36.7 °C; blood pressure, 142/60 mmHg; and heart rate, 84 beats/min. The initial blood test indicated the following: white blood cell count, 6500/μL; platelet count, 22.8 × 10^4^/μL, total protein (TP); 6.9 g/dL, C-reactive protein (CRP); 0.14 mg/dL, prothrombin time (PT), INR, 1.2; activated partial thromboplastin time (APTT), 40 s; and HbA1c, 6.0%.

Although there were no suspicious symptoms or signs suggesting the possibility of human immunodeficiency virus (HIV) infection or leukemia, we ruled out these diseases through blood examination. Because the patient presented with gingival necrosis, spontaneous bleeding, severe pain, and halitosis, he was temporarily diagnosed with NPD. Periodontal examination and scaling were not performed during the first visit to avoid exacerbating gingival pain. Instead, we carefully removed the accumulated dental plaque from the tooth surfaces using gauze and applied 2% minocycline hydrochloride ointment to the gingival mucosa and periodontal pocket throughout the oral cavity.

### 2.2. Day 7

**Figure 2:**
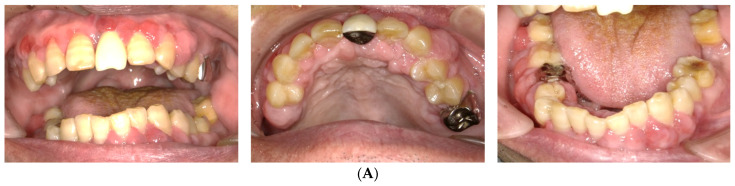

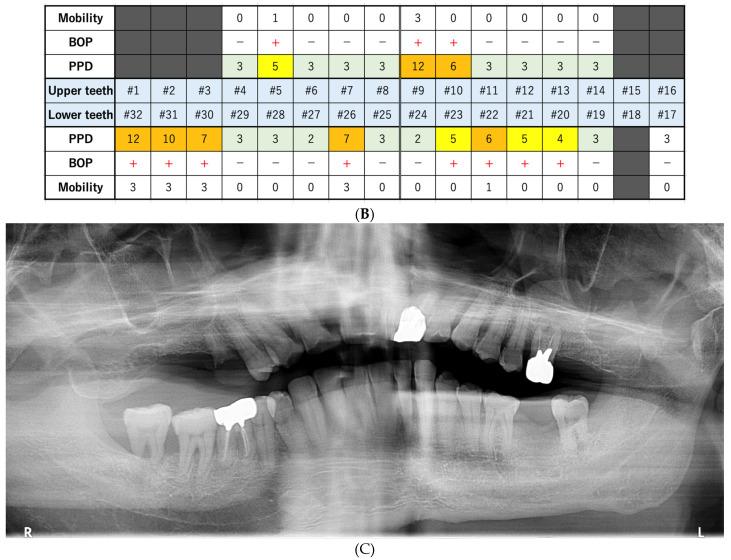
The findings at 7 days after the first visit. (**A**) These intraoral photos show that the acute symptoms, including redness, swelling, and bleeding, have decreased. (**B**) Periodontal assessment includes tooth mobility (Mobility), bleeding of probing (BOP), and probing pocket depth (PPD) (maximum value on each tooth). Black color boxes indicate missing teeth. The green color box indicates acceptable PPD (not exceeding 3 mm). Yellow color boxes indicate PPD of 4 or 5 mm. Orange color boxes indicate PPD equal to or greater than 6 mm. BOP (−) indicates no bleeding. BOP (+) indicates bleeding. (**C**) Panoramic X-ray reveals that the entire alveolar bone has horizontal absorption.

The acute symptoms resolved on day 7 (Figure 2A). We conducted a periodontal examination, including an assessment of probing pocket depth (PPD) and bleeding on probing (BOP). Because our patient had difficulty undergoing extensive examinations, we evaluated the one-point PPD instead of the six-point PPD (Figure 2B). Panoramic radiography was also performed (Figure 2C). During the periodontal examination, the entire circumference of each tooth was explored using the walking probe technique, and the value of the deepest pocket was recorded as representative of that tooth. In 11 teeth, PPD ≥ 4 mm with bleeding was detected. Of these, seven had deep pockets of >6 mm (Figure 2B). Panoramic radiography revealed a horizontal absorption of the entire alveolar bone (Figure 2C). On the basis of these comprehensive findings, NP was confirmed as the final diagnosis. Our patient also met the transiently and moderately compromised status criteria in the 2018 classification of NPD.

To improve daily oral hygiene, a team of periodontists, dentists, and dental hygienists educated care workers and other staff at the nursing home on appropriate oral cleansing, including brushing three times a day using the Bass technique. Basic periodontal therapy, including whole-mouth scaling and debridement of the root surfaces using hand and ultrasonic instruments, was also performed.

### 2.3. Day 14 and Later

**Figure 3:**
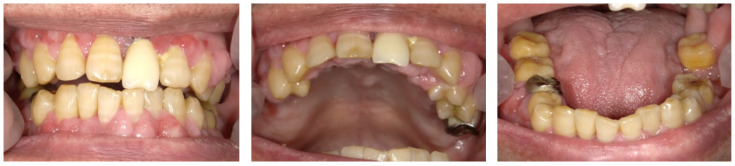
Intraoral photos at 14 days. Acute symptoms, such as gingival redness and bleeding, disappeared.

On day 14, the patient’s periodontal condition was seen to have improved following treatment, and the gingival necrosis, redness, and pain resolved (Figure 3). The halitosis had also reduced. After periodontal treatment, six teeth had a poor prognosis (#4, #9, #26, #30, #31, and #32). These teeth had severe mobility and dental caries. We discussed the issues with the patient, and extraction of these problematic teeth was planned considering the patient’s overall well-being and situation, including the general condition, previous and present medical history, medication, paralysis, nursing care system, and the patient’s needs.

**Figure 4:**
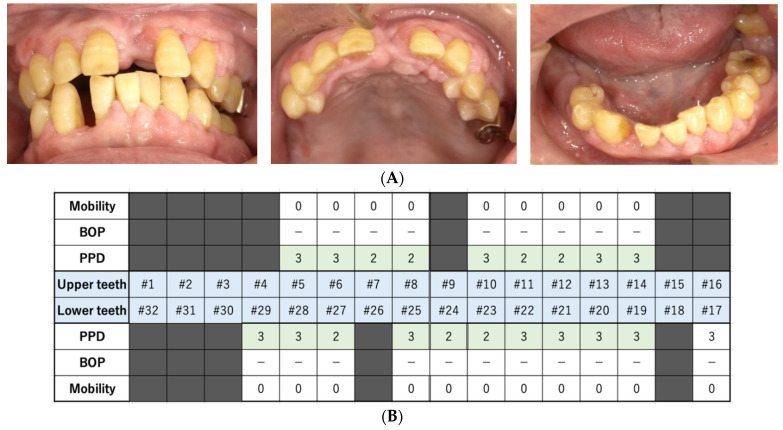

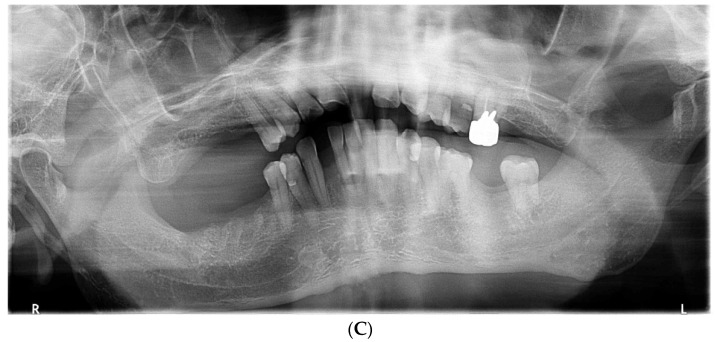
The findings at 77 days after the first visit. (**A**) These intraoral photos indicate good periodontal condition. (**B**) Periodontal assessment, including that of tooth mobility (Mobility), bleeding of probing (BOP), and probing pocket depth (PPD) (maximum value on each tooth). Black color boxes indicate missing teeth. A green color box indicates acceptable PPD (not exceeding 3 mm). BOP (−) indicates no bleeding. (**C**) Panoramic revealed that the alveolar bone resorption had not progressed.

On day 21, we extracted three teeth (#4, #9, and #32). One week later, on day 28, we extracted three more teeth (#26, #30, and #31). Seven days before the first extraction appointment, apixaban, a direct oral anticoagulant (DOAC), was changed to clopidogrel sulfate (75 mg/day), an antiplatelet agent. We thoroughly stopped the bleeding from the extraction fossa using sutures and cellulose oxide. No severe bleeding occurred after tooth extraction. The sutures were removed seven days after each extraction appointment. Seven days after the second extraction appointment, apixaban was restarted, and clopidogrel sulfate was ceased. On day 77, BOP and PPD ≥ 4 mm were no longer observed (Figure 4). Currently, dentists visit the patient fortnightly to manage his oral hygiene. To date, good oral health has been maintained.

## 3. Discussion

The diagnosis of NPD should be based on diagnostic criteria comprising typical clinical findings and the causes of immunosuppression. The clinical features of NG have been described in previous studies [10,12,13,14] and include necrosis and ulceration of the interdental papillae (94–100%), gingival bleeding (95–100%), pain (86–100%), pseudomembrane formation (73–88%), and oral halitosis (84–97%). Extraoral signs include adenopathy (44–61%) and/or fever (20–39%). In addition to the signs and symptoms of NG, NP also involves loss of periodontal attachment and bone destruction. Moreover, extraoral signs are much more likely in NP [15]. NP could be the result of one or more episodes of NG (less frequent periodontal pocket formation) or NG occurring at a site previously affected by periodontitis [4,16]. Our patient presented with gingival necrosis, ulceration of the interdental papillae, gingival bleeding, and pain during the initial examination. Radiographic examination revealed distraction of the alveolar bone. Because the patient’s presenting features were representative of NP, NP was diagnosed. 

NPD is usually found in immunosuppressed patients, such as those with HIV, leukemia, diabetes, older adults, or immunosuppressants [6,7,8,9]. Other etiological factors include tobacco smoking, malnutrition, psychological stress, and poor oral hygiene [11]. In this case, the patient showed typical symptoms of NP. His condition was compromised by advanced age and type 2 diabetes mellitus. Our patient developed NP after being transferred to a nursing home. Previously, he had lived independently. This finding suggests that strong psychological stress due to environmental changes may trigger the onset of NP. Stress alters immune responses and changes a patient’s behavior. The biological plausibility of this assumption is based on the reduction in gingival microcirculation and salivary flow, increased serum and urine levels of 17-hydroxycorticosteroid [17], changes in the functions of neutrophils and lymphocytes, and an increase in periodontal pathogens, such as P. intermedia [18]. Moreover, the patient’s oral hygiene worsened due to his physical disability after cerebral infarction, which may have enhanced the development of NP. Plaque accumulation is a predisposing factor of NPD. Limited tooth brushing due to pain may further contribute to plaque formation [10,19,20]. Taken together, our patient met the transient and middle-compromised status criteria in the 2018 classification of NPD. We believe that our patient had a low risk of developing severe necrotic conditions and estimated that our patient would achieve remission of symptoms by adapting to the current environment and basic periodontal treatment. 

Treatment of NP depends on the extent and severity of the disease. In the case of localized lesions, daily professional mechanical debridement of the teeth adjacent to the affected gingiva and topical application of an antiseptic solution, including chlorhexidine gluconate and povidone-iodine, are recommended [21,22]. Antibiotics may be used in cases of more generalized or severely painful lesions [23]. In our patient, the clinical manifestations of NP occurred over a broad area of the oral cavity, and the patient complained of severe pain. Therefore, we initially administered 2% minocycline hydrochloride using a local drug delivery system before commencing the nonsurgical periodontal therapy. This antibacterial treatment was extremely effective and dramatically improved the patient’s acute symptoms within one week. 

NP recurrence is not uncommon. Daily optimal plaque control and regular reviews are necessary to monitor signs of future diseases [3]. Our patient had difficulties in independently maintaining his oral hygiene due to his physical disability; therefore, care workers and other staff at the nursing home helped with his daily oral hygiene even after his acute symptoms resolved and basic periodontal therapy was completed. Furthermore, our dental and other medical staff visited him fortnightly to monitor and control his diabetes and psychosocial stress and review his dental health to reduce the risk of NP recurrence.

Our patient experienced severe generalized gingival bleeding during the first examination. Our patient used apixaban, a DOAC, as an anticoagulation therapy for the prevention of stroke with atrial fibrillation. DOAC may have contributed to severe bleeding. DOACs are alternatives to warfarin. The annual use of DOACs is increasing. DOACs inhibit thrombin and factor Xa. Our patients took apixaban, which targets factor Xa [24]. DOACs have a rapid onset, short half-life, and fewer drug-food interactions than warfarin [25,26]. The efficacy of DOACs is not determined by activated partial thromboplastin time (APTT) or prothrombin time (PT), although APTT or PT are usually used for coagulation screening. The pharmacodynamics of DOACs are predictable. Moreover, their administration schedules are strictly fixed. Therefore, unlike warfarin, therapeutic monitoring is not necessary [27,28]. The patient’s PT-INR and APTT, which reflect the function of extrinsic and intrinsic coagulation, respectively, were almost normal. Few reports have examined the perioperative management of patients treated with DOACs. Yoshikawa et al. reported that the incidence of post-extraction bleeding associated with DOACs is lower than that associated with warfarin and concluded that interruption of DOAC therapy is not necessary for tooth extraction if the procedure is performed at least 6 h after the last dose [24]. In contrast, other studies have reported that the incidence of postoperative bleeding in patients receiving DOAC therapy ranged from 5.5% to 40% [26,29,30,31], compared with 2% to 26% [32,33,34] in patients receiving warfarin therapy. Therefore, bleeding after tooth extraction was a concern in our patient, and close monitoring was required. In contrast to anticoagulants, antiplatelet agents are involved in primary hemostasis and do not affect the subsequent process, including secondary hemostasis or the fibrinolytic system. Therefore, once primary hemostasis is achieved, the risk of an antiplatelet agent causing post-extraction hemorrhage is lower than that of an anticoagulant [35]. Hence, we changed the patient’s anticoagulant (apixaban) to an antiplatelet agent (clopidogrel sulfate) to reduce the risk of both stroke and post-extraction bleeding.

## 4. Conclusions

We treated an elderly patient living in a nursing home for NP in cooperation with his physician and nursing staff. Our patient had mild immunosuppression due to type 2 diabetes and psychosocial stress. He was diagnosed with NP based on his typical symptoms, such as prominent bleeding and suppurative discharge from the gingiva, in addition to crater-shaped ulcerations of the interdental papillae. After basic periodontal therapy, the hopeless teeth were extracted. Consequently, our patient was able to maintain good oral health, and the recurrence of NP was prevented by educating care workers and nursing staff about appropriate oral hygiene techniques, as it was difficult for the patient to independently maintain his oral hygiene. This report describes a case of NPD in an elderly medically compromised patient with poor oral hygiene who required nursing home care and highlights the importance of managing NPD through interprofessional collaboration.

## Data Availability

For data information, contact the corresponding author.
